# Structural Identifiability of Systems Biology Models: A Critical Comparison of Methods

**DOI:** 10.1371/journal.pone.0027755

**Published:** 2011-11-22

**Authors:** Oana-Teodora Chis, Julio R. Banga, Eva Balsa-Canto

**Affiliations:** Bioprocess Engineering Group, IIM-CSIC, Vigo, Spain; Centre for Genomic Regulation (CRG), Universitat Pompeu Fabra, Spain

## Abstract

Analysing the properties of a biological system through *in silico* experimentation requires a satisfactory mathematical representation of the system including accurate values of the model parameters. Fortunately, modern experimental techniques allow obtaining time-series data of appropriate quality which may then be used to estimate unknown parameters. However, in many cases, a subset of those parameters may not be uniquely estimated, independently of the experimental data available or the numerical techniques used for estimation. This lack of identifiability is related to the structure of the model, i.e. the system dynamics plus the observation function. Despite the interest in knowing *a priori* whether there is any chance of uniquely estimating all model unknown parameters, the structural identifiability analysis for general non-linear dynamic models is still an open question. There is no method amenable to every model, thus at some point we have to face the selection of one of the possibilities. This work presents a critical comparison of the currently available techniques. To this end, we perform the structural identifiability analysis of a collection of biological models. The results reveal that the generating series approach, in combination with identifiability *tableaus*, offers the most advantageous compromise among range of applicability, computational complexity and information provided.

## Introduction

Modelling and simulation offer the possibility of integrating information, performing *in silico* experiments, generating predictions and novel hypotheses so as to better understand complex biological systems. However, the quality of the results will highly depend on the predictive capabilities of the model at hand. In this regard, the selection of an adequate modelling framework for the system under consideration and for the questions to be addressed is crucial [1] together with the capacity to anchor model sophistication with experimental data [2]. In this respect, parameter estimation by means of data fitting has become a critical step in the model building process [3].

However, and despite the ever increasing availability and quality of biological data, this parameter estimation step still remains a difficult mathematical and computational problem.

It has been argued that such difficulties are often originated in the lack of identifiability, i.e. in the difficulty or (in some cases) impossibility of assigning unique values for the unknown parameters. This has been in fact the case in many examples found in the literature [4]–[8]. These works report the impossibility to asses unique and meaningful values for the parameters since broad ranges of parameter values result in similar model predictions.

But what is the exact origin of the lack of identifiability? We can distinguish between structural and practical identifiability. Structural identifiability is a theoretical property of the model structure depending only on the system dynamics, the observation and the stimuli functions [9]. Practical identifiability is intimately related to the experimental data and the experimental noise.

Although the questions seem rather similar, there are several crucial differences. Possibly the most important has to do with the capability to recover identifiability. If some parameters turn out not to be structurally identifiable, numerical approaches will not be able to find reliable values for them. In those situations, the only possibilities for a successful model building will be i) to reformulate the model (reducing the number of states and parameters), ii) to fix some parameter values (for example, those which are less relevant to model predictions) or iii) to design new experiments by adding measured quantities (if technically possible). Lack of practical identifiability will be in general terms solvable, providing the experimental constraints allow designing sufficiently rich experiments. In this regard, recent works suggest the use of model based (optimal) experimental design to iteratively improve the quality of parameter estimates [10]–[13].

There are, at least, two reasons to asses identifiability. First, most of the model parameters have a biological meaning, and we are interested in knowing whether it is at all possible to determine their values from experimental data. Second, numerical optimisation approaches will find difficulties when trying to estimate the parameters of a non-identifiable model.

In this regard, practical identifiability analysis has received substantial attention in the recent literature. Local analyses are based on the computation of local sensitivities, the Fisher Information Matrix, the covariance matrix, or the Hessian of the least-squares function [14], [15]. Hengl et al. [16] proposed the method of mean optimal transformations to reduce the number of model parameters to improve practical identifiability. Balsa-Canto et al. [10] suggested the use of a bootstrap based approach so as to quantify practical identifiability in terms of eccentricity and pseudo-volume of the robust confidence hyper-ellipsoid. In a more recent work, the same authors suggested the use of the global rank of parameters to assess the relative influence of the parameters in the observables and to anticipate lack of structural or practical identifiability [17].

Despite the importance of knowing *a priori* whether there is any chance of uniquely estimating all model unknowns, the structural identifiability analysis has been ignored in the vast majority of modelling studies in systems biology. Only recently some works have considered the structural identifiability analysis of cell signalling related examples. Balsa-Canto et al. [17] proposed the use of power series based approaches combined with identifiability *tableaus* so as to asses the identifiability of the model of the NF
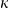
B module by Lipniacki et al. [4]; Roper et al. [18] considered the analysis of different alternative models of a single phosphorylation-dephosphorylation cycle in the MAPK cascade [19], by means of a differential algebra based approach.

However, the structural identifiability analysis for general non-linear dynamic models in systems biology is still a challenging question. Even though a number of methods exist [20], there is no method amenable to every model, thus at some point we have to face the selection of one of the possibilities.

This work presents a critical comparison of currently available methods so as to evaluate their potential in systems biology. In particular, we will consider the Taylor series method [21], the generating series method [22], both complemented with the identifiability *tableaus* [17], the similarity transformation approach [23], the differential algebra based method [24], [25], the direct test method [26], [27], a method based on the implicit function theorem [28] and the recently developed test for reaction networks [29]–[31].

The advantages and disadvantages of all these methods are evaluated on the basis of a collection of examples of increasing size and complexity. The selected models include different types of non-linear terms, such as generalised mass action (GMA), Michaelis-Menten and Hill kinetics, as typically found in systems biology models. The six different examples considered are: the Goodwin oscillator model [32], a pharmacokinetics model that describes the receptor-mediated uptake of glucose oxidase [33], the model of a glycolysis inspired metabolic pathway [34], a high dimensional non-linear model which represents biochemical reaction systems [35], the model of the central clock of *Arabidopsis Thaliana* [36] and the model of the NF
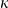
B signalling module [4].

## Methods

### Mathematical model formulation

We will assume a biological system described by:
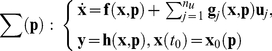
(1)


where 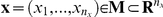 is the state variable, with 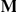 a subset of 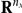 containing the initial state, 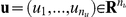 a 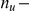
dimensional input (control) vector with 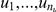 smooth functions, and 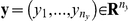 is the 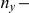
dimensional output (experimentally observed quantities). The vector of unknown parameters is denoted by 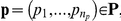 and in general is assumed to belong to an open and connected subset of 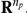 The entries of 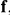 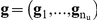 and 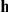 are analytic functions of their arguments. These functions and the initial conditions may depend on the parameter vector 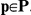


It should be noted that typical models in systems biology, such as GMA models or those incorporating Michaelis-Menten or Hill type kinetics can be easily drawn in the format of Eqn. (1).

### Structural identifiability definition

Structural identifiability regards the possibility of giving unique values to model unknown parameters from the available observables, assuming perfect experimental data (i.e. noise-free and continuous in time) [9].

- A parameter 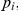 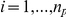 is *structurally globally (or uniquely) identifiable* if for almost any 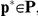

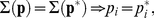
(2)
- A parameter 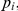 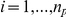 is *structurally locally identifiable* if for almost any 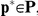 there exists a neighbourhood 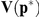 such that

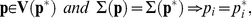
(3)
- A parameter 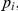 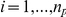 is *structurally non-identifiable* if for almost any 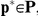 there exists no neighbourhood 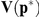 such that

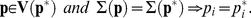
(4)

A vector 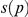 is an *exhaustive summary* of the experiment if it contains only the information about the parameters 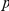 that can be extracted from knowledge of 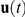 and 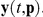


From the previous definitions, structural global (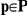) and local (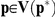) identifiability can be checked by using the exhaustive summary as follows:
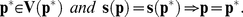
(5)


### Methods for testing structural identifiability

Structural identifiability analysis of linear models is well understood and there are a number of methods to perform such a task. In contrast, there are only a few methods for testing the structural identifiability of non-linear models: the Taylor series method [21], the generating series method [22], the similarity transformation approach [23], the differential algebra based method [24], [25], the direct test [26], [27], a method based on the implicit function theorem [28] and the recently developed test for reaction networks [29], [30].

### Taylor series approach

The Taylor series approach [21] is based on the fact that observations are unique analytic functions of time and so all their derivatives with respect to time should also be unique. It is thus possible to represent the observables by the corresponding Taylor series expansion in the vicinity of the initial state 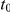 and the uniqueness of this representation will guarantee the structural identifiability of the system. The idea is to establish a system of non-linear algebraic equations in the parameters, based on the calculation of the Taylor series coefficients, and to check whether the system has a unique solution.

Let us assume that the state variables 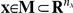, the outputs 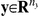, the inputs 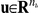 and the functions 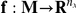 and 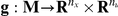 in Eqn. (1) have infinitely many derivatives with respect to time. Let us also assume that 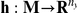 has infinitely many derivatives with respect to the state vector components and their successive derivatives. The Taylor series expansion of the observation function, in a neighbourhood of the initial state, is then given by
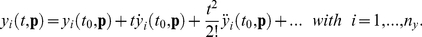
(6)


If we define:
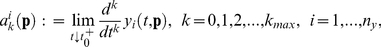
(7)


then a sufficient condition for global structural identifiability is given by
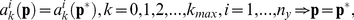
(8)


where 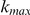 is the smallest positive integer, such that the symbolic computations give the solution of the parameters.

Possibly the major disadvantage of this method is related to the impossibility to define *a priori* the value of 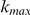, thus, in general, it will not be possible to talk about a “omplete”resolvability for the cases where 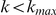. Some bounds have been established for particular types of models. For example, for a linear model the upper bound on the number of derivatives should be 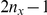 [37], for bilinear models, 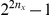 and for homogeneous polynomial systems, 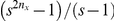, where 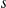 represents the degree of the polynomials [38]. For a single output model, Margaria et al. [39] showed that 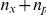 derivatives are sufficient to determine the structural identifiability using the Taylor series method. These bounds could be higher for real problems, particularly when the germ is not informative, i.e. when the Taylor coefficients become zero at the initial conditions.

Another important disadvantage of this method is that the usual complexity of the resulting algebraic parametric relations makes the analysis difficult, allowing, in many cases, only for local identifiability results [40]. This is particularly true when the number of required derivatives is large. This explains why, despite its conceptual simplicity and that computations may be simplified when the initial conditions are known, this approach has not become popular in practice [41].

### Generating series approach

Conceptually similar to the Taylor method, in the generating series approach [22] the observables can be expanded in series with respect to time and inputs in such a way that the coefficients of this series are the output functions 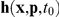, and their successive Lie derivatives along the vector fields 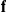 and 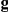 (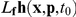, 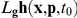, 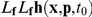, 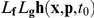, 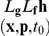, 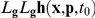 and so on).

The Lie derivative of 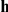 along the vector field 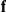, is given by:
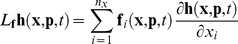
(9)


with 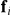 the 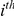 component of 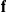,where 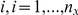.

The exhaustive summary contains the coefficients of 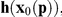 and the successive Lie derivatives along 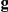 and/or 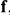 evaluated at the initial conditions 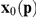. The model (1) is structurally globally identifiable if the exhaustive summary is unique.

As in the case of the Taylor approach, the major disadvantage of the generating series approach is that the minimum number of required Lie derivatives is unknown. The lack of such a bound offers only sufficient, but not necessary, conditions for identifiability. The advantage is that the mathematical expressions obtained with the generating series method are usually simpler than those obtained with the Taylor series approach [42].

It should be remarked at this point that both power series based methods may be applied to arbitrary non-linear functions 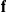, 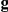 and 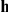 in the model (1), thus being excellent candidates to perform the analysis for the models in systems biology. However, the solution of the resultant set of non-linear algebraic equations in the parameters may be challenging (or impossible) even with the aid of symbolic manipulation software. In this concern, the systematic computation of so called identifiability *tableaus* [17] is introduced here as a way to easily visualise the possible structural identifiability problems and to systematise the solution of the resulting algebraic system of equations on the parameters.

### Identifiability *tableaus*

The *tableau* represents the non-zero elements of the Jacobian of the series coefficients with respect to the parameters. It consists of a table with as many columns as parameters and with as many rows as non-zero series coefficients (in principle, infinite).

If the Jacobian is rank deficient, i.e. the *tableau* presents empty columns, the corresponding parameters may be non-identifiable. Note that since the number of series coefficients may be infinite, structural non-identifiability may not be fully guaranteed unless higher order series coefficients are demonstrated to be zero.

If the rank of the Jacobian coincides with the number of parameters, then it will be possible to, at least, locally identify the parameters. In this situation a careful inspection of the *tableau* will help to decide on an iterative procedure for solving the system of equations, as follows:

- The number of non-zero coefficients is usually much larger than the number of parameters. In practice this means that we should select the first 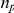 rows that guarantee the Jacobian rank condition. The *tableau* helps to easily detect the necessary coefficients and to generate a “minimum” *tableau*.
- A unique non-zero element in a given row of the minimum *tableau* means that the corresponding parameter is structurally identifiable. If the parameters in this situation can be computed as functions of the power series coefficients, they can be then eliminated from the “minimum” *tableau* to generate a “reduced” *tableau*. Subsequent reductions may lead to the appearance of new unique non-zero elements, and so on. Thus, all possible “reduced” *tableaus* should be built in sequence first.
- Once no more reductions are possible, one should try to solve the remaining equations. Since it is often the case that not all remaining power series coefficients depend on all parameters, the *tableau* will help to decide on how to select the equations to solve for particular parameters.
- If several meaningful solutions exist for a given set of parameters, then the model is said to be structurally locally identifiable.

### Similarity transformation approach

The similarity transformation approach [23] is based on the local state isomorphism theorem. The model should be locally reduced, i.e. controllability and observability conditions must be fulfilled at 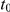 and it is assumed that the entire class of bounded and measurable functions is available for stimulus. The method seeks state variable transformations that leave invariant the stimuli-observables map and the structure of the system.

The local state isomorphism is used to establish a set of first order linear inhomogeneous partial differential equations which is used to construct the functional form of such transformations. Unfortunately, the solution of the partial differential equations may be complex, and the need to test controllability and observability conditions poses additional problems to the application of this methodology for general non-linear systems.

An alternative was proposed by Denis-Vidal and Joly-Blanchard [43] that allows to obtain direct relations of the components of the isomorphism.

The identifiability of the parameters of the model (1) can be obtained by using the local state isomorphism theorem as follows:

**Theorem 1.** [40] Let us consider the parameter values 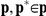 such that the model (1) is locally reduced at the initial states 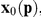 respectively 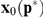 (observability and controllability rank conditions are satisfied at 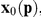 respectively 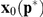), 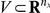 is an open neighbourhood of 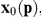 and there exists an analytical mapping 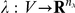 with the following properties:

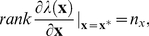
(10)

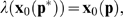
(11)

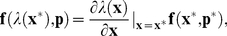
(12)

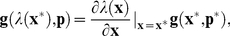
(13)

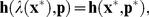
(14)


for all 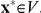 Then (1) is globally identifiable at 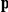 if and only if conditions (10)–(14) imply 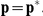


The claim of [44] is that the local state isomorphism between two state space systems corresponding to 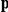 and 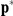 must be linear. This restriction comes from the assumption that the observability rank condition must be satisfied. Further details may be found in the recent work by Peeters and Hanzon [45]. Note that Denis-Vidal and Joly-Blanchard [43] eliminate the assumption of linearity.

The major disadvantages of this method are related to the difficulty of assessing the observability condition and the complexity to solve the differential equations (12) for general non-linear dynamic systems. Even the modifications proposed by Denis-Vidal and Joly-Blanchard [43] may not be enough for large scale highly non-linear models.

### Direct test

The conceptually simplest approach to test structural identifiability is the so called *direct test* [46], applicable to uncontrolled and autonomous systems.

This method consists basically on trying to solve directly the equality 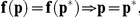 for getting local or global identifiability of the generic model (1). In general, reaching a conclusion may require excessively complicated formal manipulations or the equations to be solved may be too complicated for an analytic expression to exist, which then imposes the use of numerical methods, thus loosing the formal nature of the solution.

### Differential algebra approach

The differential algebra methods [24] are based on replacing the stimuli-observables behaviour of the system by some polynomial or rational mapping. Non-observable differential state variables are eliminated in order to get differential relations among inputs, outputs and parameters, that result from these differential relations, using Ollivier'method [47]. The exhaustive summary can be obtained and solved using algebraic methods, such as the Buchberger algorithm [48]. The algorithm is rigorous, as it converges in a finite number of steps [24].

Different strategies using the differential algebra approach have been proposed for models described by linear/non-linear differential equations, in terms of polynomial or rational functions, with or without known initial conditions.

Let us consider the general model given by (1), with 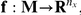 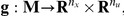 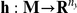 polynomial or rational functions of their arguments and the 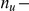
dimensional differentiable input 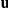. The second assumption is that the system is accessible from its initial conditions (equivalent to a “generic controllability”) [25]. The model 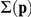 can be written as differential polynomials
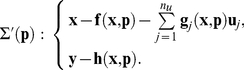
(15)


Rational systems of differential equations are reduced to the same denominator, or to a pure polynomial form.

The differential algebra approach proceeds as follows:

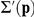
 represents the set of *differential polynomials* denoted by 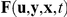.
- The *differential polynomial ring* (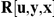) is made of polynomials of the indeterminate variables 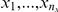 and their derivatives, the inputs 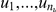 and outputs 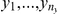 and their derivatives.

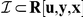
 is the ideal generated by the polynomials 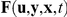 and consists of all differential polynomials that can be obtained by using addition, multiplication and differentiation. A differential ideal is called *prime* if 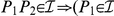 or 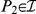).
- The differential ideal is represented by a finite basis computed by applying a set “ordering” of the variables and their derivatives, called *ranking*. In literature, the ranking is given by the inputs, as lowest ranked, outputs, and the highest rank is attributed to the state variables [24]:

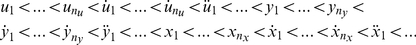
(16)The *leader* of a polynomial is the highest ranking derivative of the polynomial, and the corresponding variable is called *leading variable* [24]. The results usually change if the ranking is changed. So, we can say that differential algebra methods are rank dependent. This ranking is used to obtain an observable representation of the model, by eliminating the unmeasured state variables.

- Ritt's algorithm [49] computes the characteristic set, using the set of differential polynomials and differential ideals. With the ranking (16), the differential ideal has the characteristic set made of differential polynomials of the form

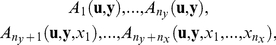
(17)where 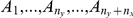 are differential polynomials, with the leaders of 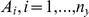 the derivatives of 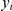. The relations (17) represent the characteristic set associated to the generic model (1) [24], [27]. The characteristic set may also be computed using the (improved) Ritt-Kolchin algorithm [50] or Rosenfeld-Gröbner algorithm [26]. All these algorithms eliminate the highest ranking variable, such that differential polynomials in 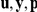 are obtained using symbolic computations. The eliminating process is called *pseudo-division*.

- Normalising the differential polynomial in 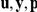 the exhaustive summary of the model is obtained. It is made of the coefficients 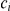 of each polynomial 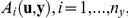 denoted by 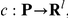 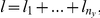 defined by 
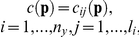
 where 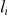 is the number of coefficients in each 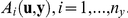 The structural identifiability is equivalent to checking the injectivity of the map 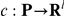. This is equivalent to solving the system of equations 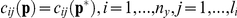 [39]. In this concern, algorithms based on the Gröbner basis may give information about the nature of the solution. Note that, in some occasions solving that system of non-linear algebraic equations may be complicated, if not impossible; for these situations it is possible to use pseudo-randomly generated numerical values instead of symbolic 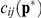 [25].

The advantage of these differential algebraic methods is that the solution of the associated algebraic equations gives precise information about the identifiability or non-identifiability of the parameters, but the disadvantage is the great computational requirements when a complex model is considered.

### Implicit Function Theorem

Proposed by Xia and Moog [28], this method is based on computing the derivatives of the observables with respect to independent variables (time) to eliminate unobserved states. A differential system is obtained, depending only on known system inputs, observable outputs and unknown parameters [41]. An identification matrix is defined, consisting of the partial derivatives of the differential equations with respect to unknown parameters. If the identification matrix is not singular, the system is said identifiable. The identifiability theory is based on the following theorem:

**Theorem 2.** [28] Let 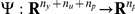 denote the function of model parameter 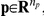 system input 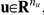 system output 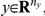 and their derivatives:
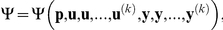



where 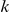 is a non-negative integer. Assume that 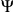 has continuous partial derivatives with respect to 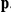 Then the generic model (1) is locally identifiable at 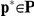 if there exists a point



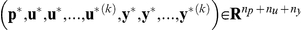
 such that
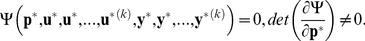
(18)


The relations in (18) are equivalent to checking structural identifiability, by examining differential polynomials 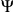 in the characteristic set, that can give us information if the model is identifiable or not, and which parameters are identifiable/non-identifiable.

This method becomes more and more complicated as the number of parameters increases due to the complexity of deriving the matrix 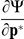. Wu et al. [41] proposed an alternative, *the multiple time points method*, that may be helpful for large scale systems. This method relies on the computation of the derivatives at a number of sampling times 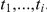 Note however, that this requires preliminary information about the observables at those sampling times.

This method offers the possibility of detecting the minimum number of observables needed to compute all parameters [28], as the computations may be performed independently for each observable.

### Identifiability analysis for dynamic reaction networks

For the case of chemical reaction networks written as in the chemical reaction network theory (CRNT) [29], [30] the structural identifiability may be checked in two steps [30]: the *reaction rate identifiability* and the *structural rate identifiability*.

The idea is to determine the structurally identifiable reaction rates, using the stoichiometric matrix, and then parameter identifiability may be computed for the considered reaction rates, using one of the above mentioned methods. In their work, Davidescu and Jorgensen make use of the generating series approach.

We consider the following facts and notations, as presented in [51]:

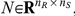
 with 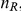 the number of reactions and 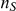 the number of species, regards the stoichiometric matrix.

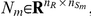
 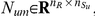 where the index 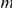 stands for measured chemical species and 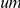 for unmeasured ones, regard the stoichiometric sub-matrix corresponding to the observed species and the stoichiometric sub-matrix corresponding to the unobserved species, respectively;
- if 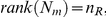 then all reactions are identifiable;
- if 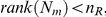 an identifiability criterion was introduced by [51], based on the difference between 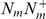 and 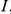 where 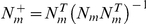 is the Moore Penrose inverse, and 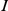 is the identity matrix.

A reaction rate is called *structurally identifiable* if the corresponding column in the matrix
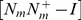
(19)


is represented by the null vector [30].

### Implementation of methods

To the authors knowledge, currently there are only two software tools available that can be used for structural identifiability analysis of non-linear models: DAISY [25] and the recently developed GenSSI toolbox [52].

DAISY implements the differential algebra based approach by using REDUCE. In principle, it is suited for any non-linear dynamic system with known numeric or symbolic non-rational initial conditions. It offers the advantage that non-expert users may perform the structural identifiability analysis even for rational models that be automatically transformed into polynomial forms. The major disadvantage is that no intermediate results may be obtained, i.e. unless the computation is completed no results will be displayed.

To surmount this difficulty, we made an implementation of the method by using the *Epsilon*, *linalg* and *Gröbner* packages, available in MAPLE, for calculations of Gröbner bases and related operations for ideals in polynomial rings. The computation of the characteristic sets has the disadvantage that one should have knowledge about the implementation and theory, and the algorithm needs to be adapted by hand, for example for rational models.

GenSSI implements the combination of the generating series approach with the identifiability *tableaus* [17]. It is also suited for non-linear dynamic models provided they are linear in the control variables (as in Eqn. (1)). It offers several advantages to non-expert users such as the possibility of handling any type of non-linear terms with transforming the models to polynomial form and the possibility of automatically incorporating known symbolic or numeric initial conditions. In addition, intermediate results on the structural identifiability of a sub-set of parameters are provided throughout the process.

The rest of the methods considered here were implemented by using suitable packages available in symbolic manipulation software tools, such as MATHEMATICA, MAPLE and MATLAB.

## Results

As mentioned before, there is no single method amenable to all types of problems for testing structural identifiability. In order to perform a critical comparison of the different possibilities in the context of systems biology, we have considered the structural identifiability analysis of the following models: the Goodwin oscillator model [32], a pharmacokinetics model that describes the receptor-mediated uptake of glucose oxidase [33], the model of a glycolysis inspired metabolic pathway [34], a high dimensional non-linear model which represents biochemical reaction systems [35], the model of the central clock of *Arabidopsis Thaliana* [36] and the model of the NF
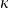
B signalling module [4].

### Case study 1: Goodwin's model

The model describes the oscillations in enzyme kinetics [53]. The state variable 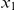 represents an enzyme concentration whose rate of synthesis is regulated by feedback control via a metabolite 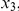 and 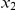 regulates the synthesis of 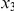. It is characterised by a rational kinetics consisting of a Hill-like term, and it is given by:
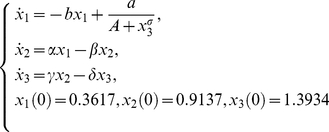
(20)


Two scenarios will be considered, (a) the typical case when only 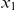 can be measured (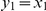) and (b) a hypothetical situation for which all states can be measured (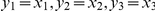).

**Figure 1:**
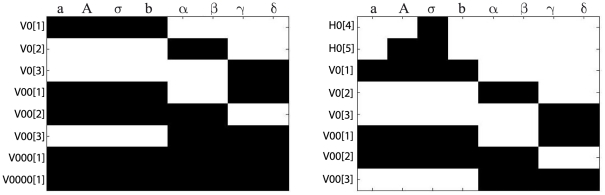
Goodwin oscillator: Identifiability *tableaus.* (a) Identifiability *tableau* obtained by means of the power series methods for the case of full observation, (b) Identifiability *tableau* obtained by means of the power series methods for the case of pure polynomial form and full observation. 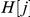 and 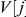 regard the different generating series coefficients, H is used for zero order coefficients whereas V correspond to the successive Lie derivatives of 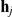 along 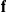, for example, 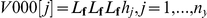. A black square in the coordinates 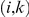 indicates that the corresponding non-zero generating series coefficient 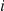 depends on the parameter 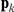.

For the case of one observable, the *power series based methods* (Taylor and generating series) were not able to compute a full rank *tableau*, because only 6 iterative derivatives could be computed. In contrast, for the case of full observation the *power series based methods* ended up in a full rank *tableau* as shown in Figure 1.(a). However, the symbolic manipulation tools were not able to solve the non-linear system of equations on the parameters, so only structural local identifiability may be assessed.

The *similarity transformation approach* could not be applied since the controllability condition is not fulfilled for this system.

The *direct test method* indicated the identifiability of 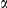, but no information was reported for the remaining parameters due to the complexity of the algebraic manipulations.

The *method based on the implicit function theorem* was only applicable for the case of full observation concluding that the remaining parameters are structurally locally identifiable provided 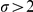.

Similarly, to apply the identifiability analysis for dynamic reaction networks we had to fix both 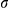 and 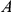 this allowing to derive the structural local identifiability of the remaining parameters.

The *differential algebra approach*, as implemented in DAISY, results in the non-identifiability of the model when 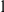 or 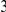 observables are considered. No results about local identifiability were reported, thus we decided to transform the original model into a full polynomial form of the model, as follows:
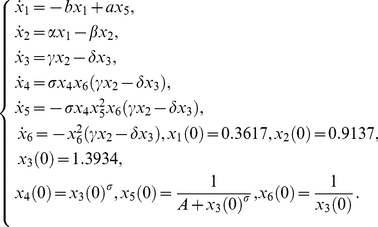
(21)


to check whether further results could be achieved.

Since algebraic operations were much simpler for this model reformulation, the power series based approaches were now able to conclude that the model (21) is structurally globally identifiable for all parameters, for the full observation case. However, the DAISY software found the model structurally non-identifiable (initial conditions not used), and was not able to finish the computations reporting errors at the time of introducing the initial conditions.

To sum up, this example illustrates how the structural identifiability analysis may contribute to the design of experiments by providing information on what to be observed so as to guarantee the structural identifiability of a given mathematical model. In addition, results also show how rational terms and Hill coefficients may pose problems to some of the methods and how pure polynomial forms may be useful so as to simplify the analysis.

For illustrative purposes, a detailed explanation of the application of the different methods to this example may be found in Supporting Information S1.

### Case study 2: Pharmacokinetics model

The pharmacokinetics model [33] is a two compartment model that embodies the ligands of the macrophage mannose receptor, and it is represented mathematically as a system of differential equations of the form:
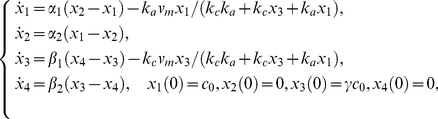
(22)


where 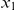 represents the enzyme concentration in plasma, 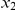 its concentration in compartment 2, 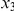 is the plasma concentration of the mannosylated polymer that acts as a competitor of glucose oxidase for the mannose receptor of macrophages, and 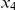 is the concentration of the same competitor in the part of the extravascular fluid of the organs accessible to this macromolecule [33]. This example is often used as a benchmark for structural identifiability methods. Two scenarios are considered (a) the case were the measured state corresponds to 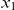 (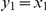), (b) the case where “an artificial output” 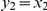 is added [54], to do so 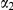 is assumed to be known [33], [35].

**Figure 2:**
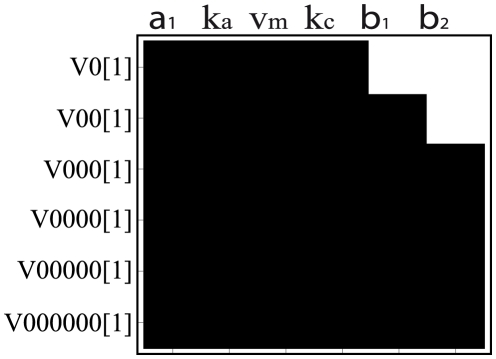
Pharmacokinetics model [**33**]. Identifiability *tableau* obtained by means of the Taylor/generating series method

The model (22) is autonomous and has no control function, so in this case the *Taylor series approach* and *generating series approach* coincide. The corresponding reduced identifiability *tableaus* are presented in Figure 2. The identifiability *tableaus* for both scenarios have full rank, thus guaranteeing, at least, structural local identifiability, even for the realistic scenario with one observable.

The introduction of a fictitious control in the model so as to fulfil the controllability condition enabled the application of the *local state isomorphism theorem* to asses local structural identifiability for the case with two observables [55]. However, the presence of a control variable does not correspond to reality, therefore the *similarity transformation approach* can not be directly applied.

The application of the *direct test method* generated two solutions for the parameters. Only for parameter 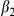 global structural identifiability was confirmed.

Saccomani et al. [35] considered the use of DAYSI for the analysis of this model concluding that for the scenario with two observables the six parameters considered are structurally globally identifiable (with known 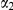). Note however that no results could be obtained for the case with one observable (with unknown 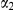), generating the computational error “heap space low”.

For the case of the application of the *implicit function theorem* it was possible to obtain the characteristic set independent of the unobserved states. However, manually generating the identifiability Jacobian matrix was too complicated. Therefore, the analysis could not be finished.

In order to apply the method for reaction networks we need to devise the network that gives rise to the model (22). For this particular example a stoichiometric matrix 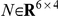 can be obtained, with the matrix of measured states 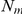 of rank 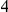. Final results assess the local identifiability of 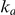, 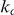 and 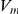. It should be noted that this may be rather complicated since the solution may not be unique [56].

From the results can then be concluded that the model is at least structurally locally identifiable for the realistic case with one observable as reported by the series based methods.

### Case study 3: Glycolysis inspired metabolic pathway

This model represents a glycolysis inspired pathway (the upper part of the glycolysis) with different physiological constraints on enzyme synthesis as described in Bartl et al. [34]. A specific enzyme, here denoted by 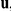 usually catalyses a metabolic reaction, expressed in terms of the stoichiometric matrix and the metabolites, here denoted by 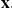 The dynamical model can be written as a system of differential equations
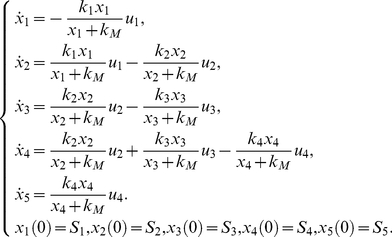
(23)


The model is considered to be fully observed, 
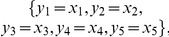
 and 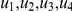 independent variables.

**Figure 3:**
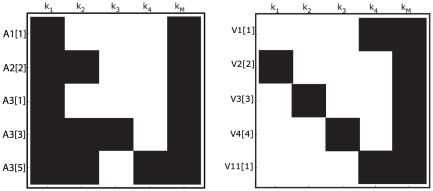
Glycolysis metabolic pathway: Identifiability *tableaus*. (a) Identifiability *tableau* obtained by means of the Taylor series method (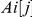, regards the 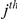 component of the 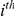 order coefficients of the Taylor series, (b) Identifiability *tableau* obtained by means of the generating series method.

The *Taylor series approach* produced an identifiability tableau of rank 5 as given in Figure 3.(a). Also, the solutions of the parameters were given: unique solution for 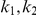 and 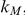 double solution for 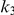 and four solutions for 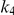. However multiple solutions were found and due to their complexity it was impossible to assess their uniqueness for the case of real positive values.

The application of the *generating series approach* indicated the global identifiability of the model. The computational cost was significantly lower as compared to the Taylor series approach. In addition, the identifiability *tableau* was not as dense, thus the solution of the system of non-linear equations on the parameters was simpler, finally resulting in an unique solution for all parameters.

The *similarity transformation approach* could not be used for this example since the observability condition is not fulfilled. The *direct test method* was also not applicable since the system is autonomous and controlled.

The *method based on the implicit function theorem* could be applied by considering the following 3 relations
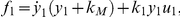


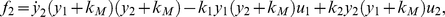


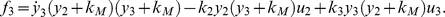



From the first equation and its derivative, the parameters 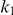 and 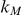 were found. Using the second one and 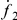, the determinant with respect to 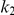 and 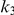 was shown to have rank 2, and from the last equation the parameter 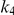 could be found. By applying Theorem 2, local identifiability was guaranteed.

Both differential algebra method implementations found the model to be globally identifiable (computation performed without the use of initial conditions).

It should be noted that the metabolic network (23) can be written in terms of stoichiometric matrix and reaction rates. The stoichiometric matrix has rank equal to 5. By choosing one matrix corresponding to the reaction rates 1, 2, 3 and 4, and then the reaction rates 1, 2, 3 and 5, and for each case applying the generating series approach, the identifiability is assessed.

Several methods (the generating series method, differential algebra and the method for reaction networks) were successful in concluding that the model is structurally globally identifiable.

### Case study 4: high dimensional non-linear model [35]

The system, that could describe a biochemical reaction network, is represented by twenty differential equations, twenty-two parameters, and all the states are assumed to be measured [35]:
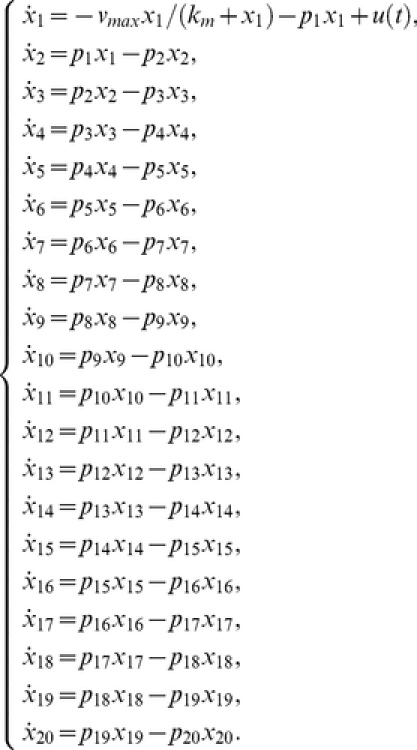
(24)


Saccomani et al. [35] considered the analysis of this system by means of the *differential algebra approach* using DAISY software. They concluded that the model is structurally globally identifiable after 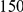 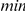 in a computer of 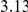 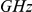 and 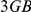 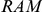.

**Figure 4:**
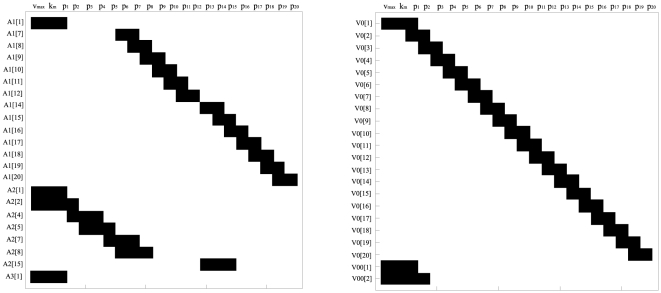
High dimensional nonlinear model: Identifiability *tableaus*. (a) Identifiability *tableau* obtained by means of the Taylor series method, (b) Identifiability *tableau* obtained by means of the generating series method.

The application of the *Taylor series approach* in combination with the identifiability *tableaus* resulted in structural global identifiability of the model in a few seconds. The reduced identifiability tableau (Figure 4.(a)) needed only 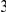 derivatives to achieve the maximum rank 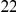. The solution of the algebraic system was given by considering the following groups of parameters: 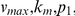 then, 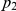 can be calculated individually. Knowing the solution of these parameters, the next group to be computed is given by 
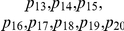
, and 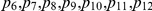. The fourth group of parameters is 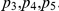 All 22 parameters have unique solution, so the model (24) is structurally globally identifiable.

The *generating series approach* in combination with the identifiability *tableaus* also concludes that the model is structurally globally identifiable. The corresponding identifiability *tableau* is represented in Figure 4.(b). All the results were computed in approximately 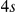 on a computer of 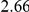 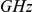 and 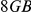 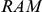.

The *similarity transformation method* requires observability and controllability rank conditions. To prove the observability rank condition we should calculate the rank of the subspace generated by consecutive differentials of 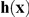 and 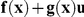. The rank 22 was obtained in MATLAB, in a few minutes, after five iterations. Unfortunately, the controllability condition could not be assessed due to computational requirements.

The *direct test* did not provide conclusive information about the identifiability of the parameters. A unique solution was obtained, but it does not comply with the structural identifiability rules, in the sense that from 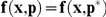, we could not find a solution 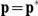, as required.

The *implicit function theorem* was successfully applied to the problem. The computations were rather simple in this case since all the state variables were measured. With an extra derivative of the corresponding output, the rank condition of the identifiability Jacobian matrix was fulfilled, and so the structural local identifiability was confirmed.

For this example, it is possible to apply the *identifiability analysis for dynamic reaction networks approach* by defining the corresponding stoichiometric matrix 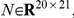 with the matrix of measured states 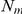 of rank 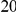. Since 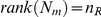 then the reaction rate identifiability is satisfied and we can directly apply the generating series approach for all reaction rates. Results coincide with the direct application of the generating series, i.e. the model is structurally globally identifiable.

The first matrix indicated the identifiability of 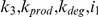. The second matrix showed the identifiability of 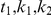; the third, 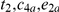; the fourth, 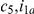 and the fifth, 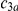.

Results obtained in this case reveal that nearly linear models with full observation are tractable for most of the methods considered. Major differences rely on the computational cost which ranges from a few seconds (GenSSI) to a couple of hours (DAISY).

### Case study 5: Arabidopsis Thaliana model

The model describes the first multi-gene loop identified in the Arabidopsis circadian clock [36] that comprises a negative feedback loop, in which two partially redundant genes, Late Elongated Hypocotyl (LHY) and Circadian Clock Associated 1 (CCA1), repress the expression of their activator, Timing of CAB Expression 1 (TOC1). A minimal mathematical representation of the system requires 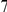 coupled differential equations and 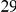 parameters. The differential equations involve Michaelis-Menten kinetics that describe enzyme-mediated protein degradation, and Hill functions that describe some transcriptional activation terms. The model is given by [36]:
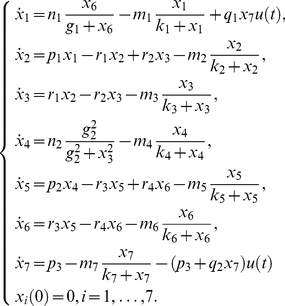
(25)


The observations correspond to the luminescence and the mRNA: 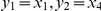 [36]. In order to analyse the role of the control variable related to the light intensity we considered the situation for which light intensity is kept constant to its maximum (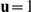) and the case corresponding to a pulse-wise light stimulation.

Results reveal that the model is not structurally globally identifiable for the case with 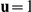 not even structurally locally identifiable since a subset of model parameters are not identifiable (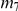, 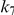, 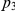, 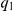 and 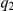).

Under the pulse-wise stimulation the *Taylor series approach*, implemented in MATHEMATICA, reached 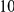 derivatives. Note that this means having only 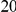 Taylor coefficients that result into a rank 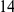 identifiability *tableau*. From the parameters appearing in the *tableau* (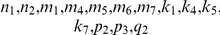) only 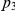 and 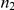 could be regarded as globally identifiable, since it was not possible to solve the system of equations for the remaining parameters. More derivatives would be required to get further results. However the task was computationally too demanding.

The *generating series approach* was able to reach the 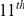 derivative resulting in an identifiability *tableau* of rank 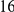. In this case a unique solution could be computed for 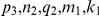. Similarly to what happened with the Taylor method, further derivatives would be required, but the task is too demanding from the computational point of view.

The *similarity transformation method* could not be applied to this example since the observability condition is not satisfied.

The *direct test method* was also not applicable since the model is controlled.

The *differential algebra approach* was not successful in providing results for this example. Both the MAPLE and DAISY implementations reported computational errors due to lack of memory.

As in previous examples, we also resorted to rewrite the model (25) in a pure polynomial form, as a system of 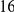 differential equations, given below:
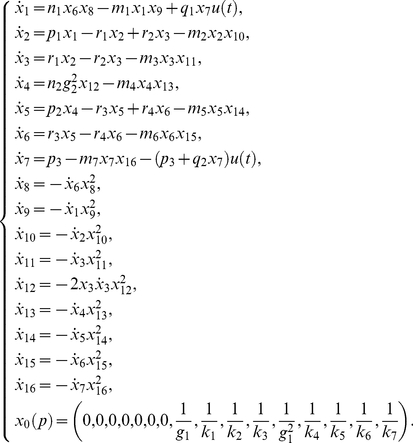
(26)


**Figure 5:**
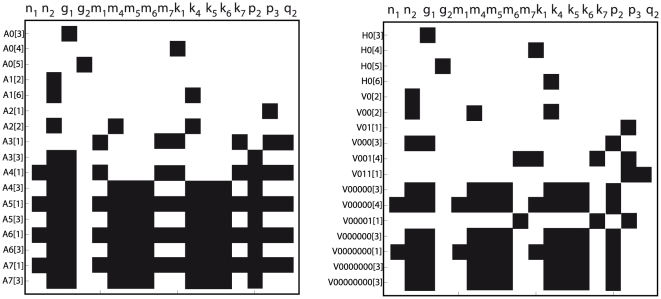
Arabidopsis Thaliana model: Reduced identifiability *tableaus*. Reduced identifiability *tableau* obtained by means of the (a) Taylor series and (b) generating series methods applied to the polynomial form of the model.

Using this pure polynomial form, and the corresponding observable states 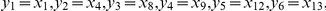 it was possible to extract more information about model identifiability. Using the Taylor series approach, we found an identifiability tableau of rank 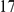, using 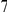 derivatives. So, at least local identifiability could be checked for the corresponding subset of parameters, as represented in Figure 5.(a). For this model formulation, uniqueness of solution was obtained for 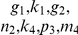.

Additional information could also be obtained using the generating series approach. The corresponding identifiability *tableau* for this method had rank 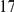, using 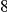 derivatives (see the corresponding reduced *tableau* in Figure 5.(b)). For this model formulation it was possible to compute unique solutions for 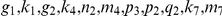. Therefore, even though pure polynomial forms result in greater computational costs, they usually provide more informative results.

**Figure 6:**
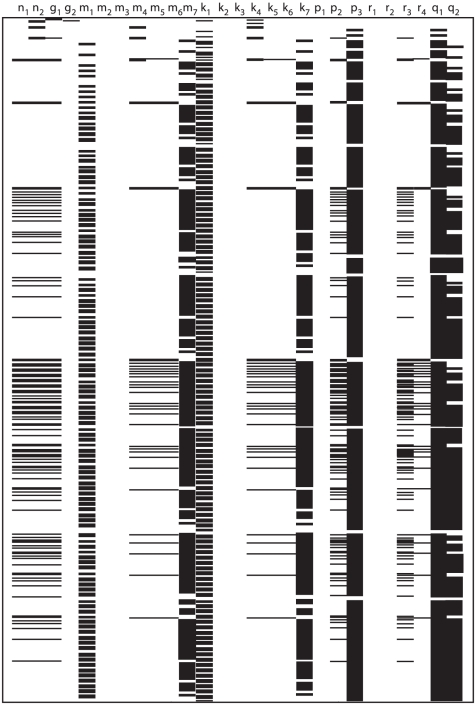
Arabidopsis Thaliana model: Full identifiability *tableau.* Identifiability *tableau* obtained by means of the generating series method applied to the polynomial form of the model. Despite the large number of terms included in the *tableau* some parameters are not appearing. The analysis may be complemented with global sensitivity analysis.

It should be noted that some parameters (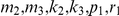 and 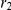) did not appear in the identifiability *tableaus* despite the large number of coefficients used in both Taylor and generating series approaches (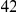 and 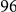, respectively). In addition, higher order coefficients were always dependent on the same parameters, as it was shown by the patterns appearing in the last rows of both *tableaus*. To further illustrate this point, the complete identifiability *tableau* obtained by means of the generating series approach is presented in Figure 6.

These results can be complemented with a global sensitivity analysis as proposed in [17]. For this example, the analysis was performed under a pulse-wise experimental scheme and the results revealed that those parameters are in fact slightly influencing the model output, thus they are expected to be structurally locally identifiable even though poorly practically identifiable.

The application of the differential algebra approach resulted in computational errors when trying to apply the initial conditions.

In order to apply the method for reaction networks the control 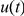 should be constant. This allows to derive a stoichiometric matrix 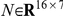 with the matrix of measured states 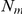 of rank 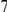. Five stoichiometric matrices of rank 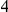 could be achieved provided we impose the condition 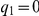. By using the generating series it is then possible to confirm the global identifiability of 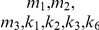 and the local identifiability of 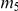 and 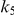. It should be noted that the method fails when trying to use the initial conditions.

The results for this case study reflect that a reduced number of observables as compared to the number of parameters poses serious problems for all methods. This will lead, in the best case, to partial solutions related to a sub-set of model parameters. In addition, as for the case of Goodwin's model, results help to decide on the type of experiment to be performed, in this case how to stimulate the system, to improve structural identifiability.

### Case study 6: NF
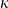
B model

The model of the NF
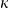
B regulatory module, as proposed by [4], is characterised by two compartment kinetics of the activators 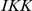 and 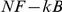, the inhibitors 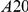 and 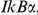 and their complexes. The model is described by the differential system:
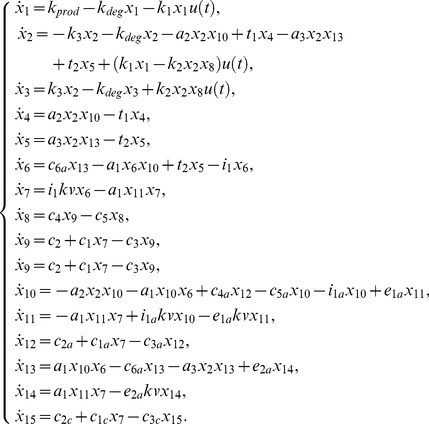
(27)


In their paper, Lipniacki et al. fixed some of the model parameters by using values from the literature. In order to assign values to the following unknown parameters:
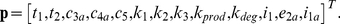
(28)


They used experimental data from previous works by Lee et al. [57] and Hoffmann et al. [58] which corresponded to the observation of 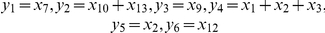.

The application of the *Taylor and generating series approaches*, with the help of the identifiability *tableaus*, to analyse the structural identifiability of the parameters in the vector 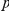 was discussed in Balsa-Canto et al. [17]. These authors found that the complexity of the equations resulting from the Taylor series approach prevented drawing conclusions on the identifiability of most of the parameters. The application of the generating series approach resulted, as expected, in a simpler system of equations. In fact it was possible to obtain as many coefficients as necessary to guarantee full rank Jacobian. In addition, the iterative solution of the set of non-linear equations resulted in the structural global identifiability of the parameters in 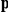.

Since the observability rank condition is not satisfied in this case, the *similarity transformation method* was not applicable. Since the system is controlled, the *direct test method* could not be applied.

The *differential algebra approach* was not successful in providing results for this example. Both implementations of the method, the one based on MAPLE and DAISY, resulted in computational errors (lack of memory problems) and were unable to calculate the characteristic set. The same reason precluded the application of the *implicit function theorem based method*.

For this example, it was possible to apply the *identifiability analysis for dynamic reaction networks approach*. The stoichiometric matrix was formed, 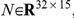 with the matrix of measured states 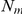 of rank 7. Five stoichiometric matrices of rank 7 were required to test the identifiability of the parameters in 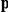. The first matrix indicated the identifiability of 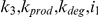. The second matrix showed the identifiability of 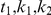; the third, 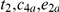; the fourth, 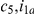 and the fifth, 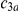.

As a summary, it can be concluded that the generating series approach, and the chemical reaction network theory combined with the generating series method, are the most suitable methods to handle generalised mass action models, particularly when the number of observables is limited and the number of derivatives required is too large for the Taylor and differential algebra methods (which are computationally not feasible for those cases).

## Discussion

The selected examples include small and medium-size models which incorporate the typical non-linear terms found in systems biology models, such as generalised mass action, Michaelis-Menten or Hill kinetics. The analysis was performed taking into account realistic measured variables (observables) available in experimental labs. For the case of the Goodwin oscillator, a hypothetical situation with full observation was also considered to illustrate how the addition of observables can improve structural identifiability.

The results (summarised in Table 1) reveal some apparent conflicting conclusions regarding the local or global identifiability of the models considered. This may be explained by taking into account that the Taylor and generating series approaches use initial conditions and symbolic quantities to solve the final algebraic system of equations on the parameters. Local identifiability is concluded when a) several solutions are found for the parameters (in the whole set of real numbers) or b) the system of equations is too complex to be fully solved. Note that in these cases local identifiability could be transformed into global identifiability when knowing the domain of definition of the parameters (for example, positive real numbers).

**Table 1:** Summary of results obtained by the different methods.

	T.S.	G.S.	S.T.	D.T.	D.A.	I.F.T.	I.D.R.N.
Goodwin one obs	NR	NR	NA	NC	NR	NA	NA
Goodwin full obs	SLI	SLI	NA	NC	SNI	SLI (σ>2)	SLI (σ, *A* fixed)
Goodwin poly. form, 1 obs	SLI	SLI	NA	NC	NR	NA	NA
Goodwin poly. form, full obs	SGI	SGI	NA	NC	SNI no i.c.	SLI no i.c.	NA
Pharma. one obs	SLI	SLI	NA	NC	NR	NR	SLI some pars.
Pharma. two obs	SLI	SLI	NA	NC	SGI	NR	NA
Glycolysis	SLI	SGI	NA	NA	SGI no i.c.	SLI	SGI
High dim. model	SGI	SGI	NR	NC	SGI	SLI	SGI
Arabidopsis clock	SLI 14 pars.	SLI 16 pars.	NA	NA	NR	NA	SLI 12 pars.
NFκB	SLI some pars.	GLI	NA	NA	NR	NR	GLI

T.S.:Taylor series approach; G.S.: generating series approach; S.T.: Similarity transformation approach; D.T.: Direct test; D.A.: differential algebra based approach; I.F.T.: method based on the implicit function theorem; I.D.R.N.: identifiability analysis based on the reaction network theory; SGI: structural global identifiable, SLI: (at least) structural local identifiable, SNI: structural non-identifiable, NA: not applicable, NC: not conclusive and NR: no results were reported due to computational errors or requirements.

Differential algebra based methods use randomly generated numerical values to handle complicated systems of equations in the parameters. Thus they may conclude global identifiability in the cases where Taylor or generating series are concluding at least local identifiability. In addition in some cases DAISY does not use initial conditions for the calculations despite their critical role in the analysis [59] being then possible that results may change from local to global. This is clearly the case when some initial conditions are zero.

Regarding a comparison of the performance of the different methods the following criteria have been used: a) range of applicability, b) computational complexity and c) information provided by the method. A general overview of the requirements, advantages and disadvantages of all methods considered is presented in Table 2.

**Table 2:** Summary of requirements, advantages and disadvanges for all methods.

**T.S.**	Requirements	- **f** *;* **g** *;* **h** may be non-linear with any dependency on **u**
		- **x** *;* **y** *;* **f** *;* **g** *;* **h** allow for infinite derivatives w.r.t. time/states
	Advantages	- conceptually simple
		- enhanced performance with identifiability *tableaus*
	Disadvantages	- unknown number of required derivatives
		- computationally demanding for low number of observable or when the initial conditions are not informative
**G.S.**	Requirements	- **f** *;* **g** *;* **h** may be non-linear but linear dependency on **u**
		- **x** *;* **y** *;* **f** *;* **g** *;* **h** allow for infinite derivatives w.r.t. time/states
	Advantages	- conceptually simple
		- simpler algebra and less computational cost than T.S.
		- enhanced performance with identifiability *tableaus*
		- software available (GenSSI)
	Disadvantages	- unknown number of required derivatives
		- computationally demanding for low number of observables or when the initial conditions are not informative
**S.T.**	Requirements	- linear dependence on **u** that must be bounded and measured
		- controllability and observability conditions
	Advantages	- software available for part of the analysis
	Disadvantages	- results in a complicated set of partial differential equations
		- computationally demanding
**D.T.**	Requirements	- uncontrolled systems
	Advantages	- conceptually simple
	Disadvantages	- requires complicated algebraic manipulations
		- computationally demanding
**D.A.**	Requirements	- **f** *;* **g** *;* **h** polynomial or rational and **u** differentiable
		- generic controllability
	Advantages	- software available (DAISY)
		- conclusive non-identifiability
	Disadvantages	- rational models are to be reduced to polynomial form
		- computationally demanding
		- limited performance when the number of observables is low
**I.F.T.**	Requirements	- **f** *;* **g** *;* **h** non-linear, differentiable and **u** differentiable
	Advantages	- characteristic set may be obtained with existing software
	Disadvantages	- complicated identifiability matrix
		- limited performance when the number of observables is low
**I.D.R.N.**	Requirements	- chemical reaction networks
		- combined with other methods
	Advantages	- analysis by groups of reaction rates
		- computationally simple
		- efficiency in combination with generating series (G.A.)
	Disadvantages	- only suitable for chemical reaction networks
		- reaction rates needed for identifiability analysis

The *Taylor series approach* is probably the most general method since it can be applied to any type of non-linear model. It is also conceptually simple as it relies on the uniqueness of a Taylor expansion of the observables around 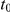. Thus the implementation and the application of the method do not require advanced mathematical knowledge. Its major drawback is that the number of required derivatives is generally unknown and it may become rather large particularly for the cases where the number of observables is small as compared to the number of parameters. In addition, final algebraic symbolic manipulations can become too complicated when solving the resulting systems of equations in the parameters. Even though, this may be partially solved by means of the identifiability *tableaus*, for some particular examples the method may be ultimately unable to provide exact information on the local/global identifiability of the parameters.

The *differential algebra based method* is based on the definition of the observables dynamics as functions of the observables by manipulating the original model. Possibly the major advantage with respect to series based methods is that it is conclusive for structurally non-identifiable models. Even though advanced mathematical skills are required so as to understand and implement the method, the recently developed DAISY software [25] enables its application to non-expert users. The major drawbacks appear in the analysis of models incorporating Michaelis-Menten and Hill kinetics, even when transforming the models to pure polynomial forms as suggested by Margaria and coworkers [39]. In addition, the method presents serious difficulties when the number of observables is low as compared to the number of parameters and the computation of the characteristic polynomial requires high order derivatives.

The applicability of the *similarity transformation approach* relies on the verification of the observability and controllability conditions and the local state isomorphism theorem. Despite many mathematical packages incorporate functions to check the observability and controllability of a given model, in home implementations are required to verify the local state isomorphism conditions. In addition, in many cases, such as most of the examples considered in this contribution, the observability condition may not be fulfilled or the associated computational burden may be too large thus precluding its application. Additional difficulties might arise when trying to analytically solve the differential equations (10)-(14).

The *direct test method* is only applicable to autonomous and uncontrolled systems. Although it is conceptually the simplest approach, for the examples considered, no reliable results could be achieved due to the complexity of the associated algebraic manipulations.

The *implicit function theorem based method* is, in principle, applicable to any differentiable. As for the case of the differential algebra approach, the method relies on the derivation of the characteristic polynomial. Thus, its complexity grows rapidly when the number of observables is low as compared to the number of parameters. In addition, it only provides information about local identifiability.

The *CRNT based method* is applicable to models that can be written in the CRNT form. This may be difficult for some particular cases with Michaelis-Menten or Hill kinetics or when the corresponding reaction network is unknown (as in some examples considered here). Results rely on the application of another identifiability analysis method, in particular the use of the generating series approach enhances the overall efficiency of the method.

The *generating series approach* in combination with the identifiability *tableaus* offers the most advantageous compromise regarding applicability, computational complexity and information provided. Its computational requirements are significantly lower than the Taylor or the differential algebra approaches, and the information provided is often more precise. This is mainly due to the following facts: i) the required number of derivatives is usually lower than for the other methods and ii) the identifiability *tableaus* are sparser, meaning that the system of non-linear equations on the parameters is simpler, thus providing more information to distinguish between local and global identifiability. The recently developed toolbox GenSSI [52] eases the application of this methodology, offering access to intermediate results throughout the process and allowing for the easy incorporation of known numeric or symbolic initial conditions to the analysis.

Since the structural identifiability analysis will be embedded in a larger systems biology work flow, the selection of the most adequate approach for the model under consideration will be critical. In this concern, we would suggest the use of the generating series approach in combination with the identifiability *tableaus* as implemented in GenSSI [52] exploiting the CRNT structure when possible. To get conclusive results on the possible structural non-identifiability of a sub-set of parameters for a given model the use of DAISY is suggested. The use of the Taylor approach is only recommended for those rare cases where control dependence is non-linear. Unfortunately remaining methods seem not be adequate to handle typical systems biology models.

### Conclusions

The unique identification of parameters in systems biology models is a very challenging task. The problem becomes especially hard in the case of large and highly non-linear models. In fact, in some cases it will be impossible to compute a unique value for the parameters independently of the available experimental data. This is particularly true for models where the ratio between the number of observables and the number of parameters is low, or when complicated non-linear terms, such as Michaelis-Menten or Hill kinetics, are present. This frequently results in a lack of structural identifiability, which is therefore a key property of these models.

In this work, we have presented a critical comparison of the available techniques for the analysis of structural identifiability of non-linear dynamic models by means of a collection of models related to biological systems of increasing size and complexity.

Results reveal that the combination of the generating series approach with identifiability *tableaus* [17] offers the best compromise between range of applicability, computational complexity and information provided.

## Supporting Information

Supporting Information S1 **Details on the application of the structural identifiability methods for Goodwin's model.**
(PDF)
