# Supplementary material for: Structural Identifiability of Systems Biology Models: A Critical Comparison of Methods
Source: PLoS One. 2011 Nov 22;6(11):e27755. doi: 10.1371/journal.pone.0027755 (PMC3222653; doi:10.1371/journal.pone.0027755)
Supplement: Supporting Information S1 — Details on the application of the structural identifiability methods for Goodwin's model. (PDF) [file pone.0027755.s001.pdf]

Supporting Information

Details on the application of the structural identifiability methods for Goodwin's model

As commented in the main text, this example is used to illustrate how the structural identifiability analysis may contribute to the design of experiments by providing information on what to be observed so as to guarantee the structural identifiability. In this regard two different situations were considered: first, the realistic case with one observable and second, a hypothetical case with full observation. Here we present in more detail how the different methods were applied to each of both scenarios.

Taylor and Generating series approaches

As the Goodwin model is autonomous and uncontrolled, the Taylor and generating series approaches generate the same result. Structural identifiability using *Taylor series approach* was verified using the identifiability *tableau.*

In the realistic case of one measured state variable, MATHEMATICA could not achieve a full rank Jacobian, because only 6 derivatives were generated, the computation of iterative derivatives being very complex. Therefore, in this scenario no conclusions may be driven.

In the case when the model is fully observable, a rank 8 identifiability *tableau* is generated, so the Goodwin model is at least structurally locally identifiable. The solution/solutions could not be computed because of the complex algebraic equations, so we cannot say anything about the uniqueness of the solution of the parameters. The corresponding identifiability *tableau* is presented in Figure 1 in the main text.

Similarity transformation approach

Similarity transformation approach is quite a restrictive method. First of all, the model should be locally reduced, that means that observability and controllability rank conditions should be satisfied. For Goodwin's model, the controllability rank condition is not satisfied neither in the case with 3 outputs, nor in the case with 1 output.

Direct test

This method relies on the truthfulness of the following relation $\mathbf{f}(\mathbf{p}) = \mathbf{f}(\mathbf{p}^*) \Rightarrow \mathbf{p} = \mathbf{p}^*$. For the Goodwin's model we consider the algebraic relations in the parameters given by:

$$\begin{array}{l} -bx_1 + \frac{a}{A+x_3^{\sigma}} = -b^*x_1 + \frac{a^*}{A^*+x_3^{\sigma^*}}, \\ \alpha x_1 - \beta x_2 = \alpha^* x_1 - \beta^* x_2, \\ \gamma x_2 - \delta x_3 = \gamma^* x_2 - \delta^* x_3. \end{array} \tag{1}$$

The relations between the parameters $a, A, \sigma, b, \alpha, \beta, \gamma, \delta$ and $a^*, A^*, \sigma^*, b^*, \alpha^*, \beta^*, \gamma^*, \delta^*$ are the following:

$$
\begin{aligned}
\beta^* &= -\frac{-\alpha^* x_1 + \alpha x_1 - \beta x_2}{x_2}, \quad \gamma^* = -\frac{-\delta^* x_3 - \gamma x_2 + \delta x_3}{x_2}, \\
a^* &= -\frac{-b^* x_1 A^* A - b^* x_1 A^* e^{ln(x_3)\sigma} - b^* x_1 e^{ln(x_3)\sigma^*} A - b^* x_1 e^{ln(x_3)\sigma^*} e^{ln(x_3)\sigma}}{A + e^{ln(x_3)\sigma}} \\
&+ \frac{b x_1 A^* A + b x_1 A^* e^{ln(x_3)\sigma} + b x_1 e^{ln(x_3)\sigma^*} A + b x_1 e^{ln(x_3)\sigma^*} e^{ln(x_3)\sigma} - a A^* - a e^{ln(x_3)\sigma^*}}{A + e^{ln(x_3)\sigma}}, \\
b^* &= b^*,\ A^* = A^*,\ \sigma^* = \sigma^*,\ \alpha^* = \alpha^*,\ \delta^* = \delta^*.
\end{aligned}
$$

From these relations it could be concluded that no parameter is identifiable. This method is conceptually simple and can be easily implemented in a typical symbolic manipulation package (for example MAPLE). However, as for this example, it is often not conclusive and some other techniques should also be used to complement the results.

Differential algebra approach

Goodwin's model is characterised by a rational term. However, as stated in the main text the models should be transformed to polynomial form so as the differential algebra approach to be applicable. We now describe the procedure to apply the method with the two different software implementations we used.

1. To use CharSets package and Epsilon in MAPLE, we have to manually transform the original model into its polynomial form. In addition, we had to fix the value of σ since the program generates the error "input must be differential polynomial" when using the general expression x_3^σ. The value $\sigma = 10$ is selected [1].
2. DAISY performs the transformation to polynomial form automatically by means of specific functions from REDUCE. However, it is unclear how σ is being treated.

For Goodwin's model with 1 and 3 outputs and when all parameters are considered, MAPLE generates an error and DAISY results in structural non-identifiability. If $\sigma = 10$, DAISY generated structural global identifiability, without the use of initial conditions when all the state variables are measured, and could not finish the computations if only the first state variable is observed.

Both tools required a relatively large computational effort to perform the analysis.

Implicit function theorem approach

The application of the method to the Goodwin oscillator with 3 state variables and one observable results in the impossibility of finding the function Ψ, depending only on the measured variables.

For the case of full observation we get the following differential polynomials

$$
\begin{aligned}
f_1 &= \dot{y}_1(A + y_3^\sigma) - a + y_1 b(A + y_3^\sigma), \\
f_2 &= \dot{y}_2 - \alpha y_1 + \beta y_2, \\
f_3 &= \dot{y}_3 - \gamma y_2 + \delta y_3.
\end{aligned} \tag{2}
$$

In the first relation only 4 parameters are involved, so we have to consider up to the third derivative of f_1. The matrix constructed with the elements $\frac{\partial f_1^{(i)}}{\partial p_1}$, $i = 0, 1, 2, 3$ and $p_1 \in \{a, A, b, \sigma\}$ may be defined only for $\sigma > 2$ and that its determinant is non zero, thus $p_1 \in \{a, A, b, \sigma\}$ are structurally identifiable. f_2 and $f_2^{'}$ are used to determine α and β. These parameters are structurally identifiable as the rank of the

corresponding Jacobian is 2. The parameters γ and δ are also structurally identifiable as the Jacobian of y_3 and $\dot{y}_3$ with respect to these parameters is 2. So, according to Theorem 2, the Goodwin's model with 3 outputs is structurally locally identifiable. It should be noted at this point that the analysis had to be performed by hand, since no symbolic package would compute derivatives for unknown σ and that the model results structurally locally identifiable provided $\sigma > 2$.

Identifiability analysis for dynamic reaction networks

We apply this method, by considering $\sigma = 10$ and $A = 1$ [2], as the method is applicable only for the models that are encoded in the chemical reaction network theory described by mass action kinetic networks. For each component x_1, x_2, x_3, we formulate the dynamic mass balance as given in Table 1, representing the corresponding stoichiometric matrix.

Table 1. Stoichiometric matrix for Goodwin's model

	x_1	x_2	x_3
r_1	1	0	0
r_2	0	1	0
r_3	0	0	1
r_4	-1	0	0
r_5	0	-1	0
r_6	0	0	-1

We first consider the case of full observation. The matrix corresponding to the measured reaction rate, N_m, coincides with the stoichiometric matrix and the matrix corresponding to unmeasured species N_{um} is the null matrix. The matrix N_m has rank 3, and so only 3 reaction rates are considered for study each time, the process being repeated until all parameters are incorporated. We consider 2 steps, including the reaction rates r_1, r_2, r_3, and r_4, r_5, r_6. The matrix formed with the first group reaction rates, N_{m1}, is the identity matrix, and the corresponding column of each reaction rate in the matrix $[N_m N_m^+ - I]$ is zero, so they are structurally identifiable. The parameters included in r_1, r_2, r_3 are a, α, γ, that are determined from the first order Lie derivatives, the other parameters being considered fixed. The second rates group has the matrix $N_{m2} = -I_3$. Also in this case the column corresponding to each reaction rate r_4, r_5, r_6 in $[N_m N_m^+ - I]$ is zero, ensuring the structural identifiability. The parameters involved in these reaction rates are b, β, γ and were found identifiable from the first order Lie derivatives with respect to the outputs.

If just one output is considered, the stoichiometric matrix is split into the matrix of measured species, N_m, and the one of the unmeasured species, denoted by N_{um}, given by:

$$N_m = \begin{pmatrix} 1 \\ 0 \\ 0 \\ -1 \\ 0 \\ 0 \end{pmatrix}, \quad N_{um} = \begin{pmatrix} 0 & 0 \\ 1 & 0 \\ 0 & 1 \\ 0 & 0 \\ -1 & 0 \\ 0 & -1 \end{pmatrix}.$$

In this case the condition $N_m N_m^T$ has zero determinant and the method can not be applied.

Analysis by transforming the model to pure polynomial form

To reduce the complexity of the calculus, we considered a simpler equivalent formulation of the Goodwin's model following the advice of Margaria et al. [3]. We show here that this may be helpful in

some cases. However, this may not be generalised, since a pure polynomial form may become highly non-linear leading to computational problems.

Let's now consider the following transformations $x_4 := x_3^\sigma$, $x_5 := \frac{1}{A+x_4}$, and $x_6 := \frac{1}{x_3}$. The model becomes:

$$\begin{cases} \dot{x}_1 = -bx_1 + ax_5, \\ \dot{x}_2 = \alpha x_1 - \beta x_2, \\ \dot{x}_3 = \gamma x_2 - \delta x_3, \\ \dot{x}_4 = \sigma x_4 x_6(\gamma x_2 - \delta x_3), \\ \dot{x}_5 = -\sigma x_4 x_5^2 x_6(\gamma x_2 - \delta x_3), \\ \dot{x}_6 = -x_6^2(\gamma x_2 - \delta x_3). \end{cases} \quad (3)$$

For this formulation parameters are $a, b, \sigma, \alpha, \beta, \gamma, \delta$ and initial conditions correspond to $x_0(p) = (0.3617, 0.9137, 1.3934, (1.3934)^\sigma, \frac{1}{A+(1.3934)^\sigma}, 0.7176)$.

The scenario with one observable corresponds to the case of measuring x_1 and x_5. With the given $x_0(p)$ the model is found structurally locally identifiable using the generating series approach. Using differential algebra no results were obtained. When using initial conditions the "$1\backslash(A+(1.3934)^\sigma)$ invalid as kernel" error is displayed. The direct test concluded the identifiability of σ. The method based on the implicit function theorem can not be applied due to the impossibility to compute Ψ.

For the full observation case ($y_1 = x_1$, $y_2 = x_2$, $y_3 = x_3$, $y_4 = x_4$, $y_5 = x_5\, y_6 = x_6$) the same results are obtained using differential algebra - structurally non-identifiable (without the use of initial conditions) and invalid kernel error (when initial conditions are considered). Using power series methods the model (3) generates complete identifiability *tableaus* of rank 8 (see Figure S1) ensuring structurally global identifiability. Remark that the identifiability results differ from the ones obtained with DAISY. This may be explained by noting that differential algebra methods do not automatically incorporate initial conditions. The direct test concluded the identifiability of σ (note that the direct test results are independent of the observation function). The method based on the implicit function theorem results in the local structural identifiability of the model.

It should be remarked that different polynomial forms of originally rational models may lead to different solutions, at least when using the differential algebra approach. It is also important to note the critical role of the initial conditions in the analysis, since this may lead to wrong conclusions.

Figure S1. Goodwin model - polynomial form: (a) Identifiability tableau for (3) (one observable) (b) Identifiability tableau for (3) (full observation)

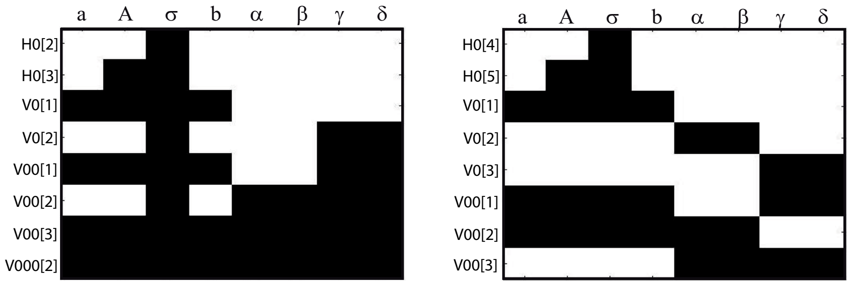

Figure S1. (a) Identifiability tableau for (3) (one observable) (b) Identifiability tableau for (3) (full observation)

References

1. Balsa-Canto E, Peifer M, Banga J, Timmer J, Fleck C (2008) Hybrid optimization method with general switching strategy for parameter estimation. BMC Systems Biology 2:26, DOI:10.1186/1752-0509-2-26.

2. P Ruoff CM M Vinsjevik, Rensing L (2001) The goodwin model: Simulating the effect of light pulses on the circadian sporulation rhythm of neurospora crassa. J Theor Biol 209: 29–42.

3. Margaria G, Riccomagno E, Chappell M, Wynn H (2001) Differential algebra methods for the study of the structural identifiability of rational function state-space models in the biosciences. Mathematical Biosciences 174: 1-26.
